# Half dose sugammadex combined with neostigmine is non-inferior to full dose sugammadex for reversal of rocuronium-induced deep neuromuscular blockade: a cost-saving strategy

**DOI:** 10.1186/s12871-017-0348-9

**Published:** 2017-04-11

**Authors:** Marie T. Aouad, Waseem S. Alfahel, Roland N. Kaddoum, Sahar M. Siddik-Sayyid

**Affiliations:** Department of Anaesthesiology, American University of Beirut Medical Center, P.O. Box 11–0236, Beirut, Lebanon

**Keywords:** Neuromuscular blockade, Sugammadex, Rocuronium

## Abstract

**Background:**

Sugammadex reverses the effect of rocuronium more rapidly and effectively than neostigmine, at all levels of neuromuscular blockade (NMB). However, its cost is prohibitive. The combination of half dose sugammadex with neostigmine would be non-inferior to full dose sugammadex for the reversal of deep NMB. This approach would reduce the cost of sugammadex while preserving its efficacy.

**Methods:**

Patients were randomly allocated to receive sugammadex 4 mg/kg (Group S) or sugammadex 2 mg/kg with neostigmine 50 μg/kg and glycopyrrolate 10 μg/kg (Group NS) for reversal of rocuronium deep NMB. The primary outcome was the percentage of patients who recovered to 90% Train of Four (TOF) ratio within 5 min. The non-inferiority margin was set at 10%.

**Results:**

Twenty eight patients were enrolled in each group. The number of patients who reached 90% TOF ratio within 5 min was 27 out of 28 (96%) in group S versus 25 out of 28 (89%) in group NS by intention-to-treat (difference: 7%, 95% CI of the difference: −9% to 24%). The number of patients who reached 90% TOF ratio within 5 min was 26 out of 26 (100%) in group S versus 23 out of 25 (92%) in group NS by per-protocol (difference: 8%, 95% CI of the difference: −6% to 25%).

**Conclusions:**

Sugammadex 2 mg/kg with neostigmine 50 μg/kg was at worst 9% and 6% less effective than sugammadex 4 mg/kg by intention-to-treat and by per-protocol analysis respectively. Hence, the combination is non-inferior to the recommended dose of sugammadex.

**Trial registration:**

Clinicaltrials.gov NCT 02375217, registered on February 11, 2015

## Background

Sugammadex is a new reversal agent that works differently than cholinesterase inhibitors. It binds with high affinity to rocuronium or vecuronium in the blood, decreasing its plasma concentration and creating a gradient between the plasma and the neuromuscular junction. This process results in removing the non-depolarizing agent form the receptors as it follows the concentration gradient towards the blood [1]. This novel reversal agent has changed established practices and resolved many clinical dilemmas, but has also created new challenges. It has been shown that within 5 min, 98% of patients would recover a train of four (TOF) ratio of 0.9 from a moderate rocuronium blockade (TOF of 2) with sugammadex versus only 11% of patients with neostigmine [2]. Sugammadex, if given in appropriate doses, has the ability to reverse the effect of rocuronium more rapidly and effectively than neostigmine, especially from deeper levels of neuromuscular blockade (NMB) [3]. Therefore, the availability of sugammadex has created a recent trend in the literature that advocates for the maintenance of deep levels of muscle relaxation till the end of surgery, mainly in laparoscopic surgery [4], yet the evidence on its beneficial effects is limited [5]. The claimed advantages of this practice are decreased postoperative shoulder pain [6] and improved surgical conditions [4]. Other advantages of sugammadex are the ability to reverse deep NMBs in unanticipated short surgeries without disrupting busy operating room schedules.

The superiority of sugammadex has even been established for shallower NMBs [7, 8]. A dose of 0.22 mg/kg suggamadex and 34 μg/kg neostigmine accelerates recovery from a TOF ratio of 0.5 to a TOF ratio of at least 0.9 in an average of 2 min [9]. However, the high cost of this drug is prohibitive and is a significant limitation for its routine use in many institutions, especially when relatively high doses are required. For the reversal of deep NMBs, defined as TOF = 0 [10], the use of 4 mg/kg is recommended. We hypothesize that the combination of neostigmine with half dose sugammadex (2 mg/kg) would be non-inferior to the 4 mg/kg recommended dose in reversing rocuronium-induced deep NMB. Given the high cost of sugammadex, this multimodal approach would be an effective cost saving strategy, while preserving the advantages of this novel agent. Our primary outcome was the number of patients who reverse from a TOF count of 0 to a TOF ratio of 0.9 within 5 min. Time of reversal to TOF ratio of 0.9, patients with residual neuromuscular blockade defined as TOF less than 0.9 within 10 min, time to extubation, TOF ratio at 5 min, blood pressure and heart rate after reversal administration and number of sugammadex vials used were considered as secondary outcomes.

## Methods

After obtaining the approval of the Institutional Review Board and written informed consent from patients, we conducted our study at the American University of Beirut Medical Center, Lebanon. The study was registered with the www.clinicaltrials.gov protocol registration system, principal investigator MTA, on February 11, 2015 (NCT 02375217). CONSORT statement for reporting non-inferiority and equivalence trials were followed. Inclusion criteria were patients aged 18 to 70 years, of ASA classification I–III, undergoing elective surgery and requiring the use of muscle relaxants throughout the surgery. Exclusion criteria were the presence of renal dysfunction defined as creatinine > 1.2 mg/dL or known hepatic disease, obstructive sleep apnea, allergy to rocuronium or sugammadex, pregnant or obese patients (BMI >35 kg/m^2^), patients receiving medications known to interfere with the neuromuscular transmission, or having neuromuscular diseases.

Premedication with diazepam 5 mg PO was given, standard monitors were applied and anesthesia was induced using midazolam 1–2 mg, lidocaine 1.5 mg/kg, propofol 2 mg/kg, fentanyl 100 μg and rocuronium 0.6 mg/kg to facilitate tracheal intubation. Maintenance was achieved using sevoflurane 2%, incremental doses of fentanyl and dexmedetomidine 0.5 mcg/kg/h. Dexmedetomidine was maintained till 90% recovery of TOF to avoid hand movements.

The depth of the NMB was monitored by means of kinemyography using a nerve stimulator attached to the proximal medial aspect of the forearm along the run of the ulnar nerve (GE Datex Ohmeda, M-NMT Module, Healthcare Finland Oy Helsinki, Finland). After the administration of propofol and before the administration of rocuronium, automatic calibration of the device was done and the supramaximal current of stimulation was set. TOF stimuli at the supramaximal current were applied at 20 s interval. The hand was lying freely on the arm board and kept warm by using a forced-air warming blanket. Rocuronium was given by incremental boluses of 10 mg to maintain deep NMB (TOF count = 0) [10] till the end of surgery.

A research assistant randomly assigned patients to one of two groups according to computer generated table of random numbers. The group assignment was kept in opaque sealed envelopes which were opened sequentially before study drug administration. Study drugs were drawn in 2 separate syringes, were diluted to a total volume of 10 ml with normal saline and were labeled with the randomization number. The anesthesiologist collecting the data and administering the reversal was blinded to group allocation. At the end of surgery, patients in group NS received sugammadex 2 mg/kg and neostigmine 50 μg/kg with glycopyrrolate 10 μg/kg and patients in group S received sugammadex 4 mg/kg and 10 mL of normal saline. The times from reversal administration to 90% recovery of TOF ratio, and to extubation were recorded. The number of patients who recovered 90% TOF ratio within 5 min was computed. Systolic blood pressure (SBP) and heart rate (HR) were recorded before, and after 1 and 5 min of reversal administration. The number of vials of sugammadex used was recorded. Patients who did not achieve 90% TOF ratio recovery within 10 min from study drug administration were identified as patients with residual blockade. For those patients, it was planned to proceed with unblinding and to supplement with additional sugammadex 2 mg/kg for patients in group NS. In the post anesthesia care unit (PACU) the reoccurrence of signs of residual neuromuscular blockade (rNMB) including nystagmus, laryngospasm, weakness, inability to sustain head lift, uncoordinated movements, or desaturation (SpO_2_ < 94%) was monitored by one of the investigators who was blinded to group allocation. All patients were observed for at least one hour following which they were discharged if they met standard discharge criteria.

### Statistical Analysis

Our primary outcome was the proportion of patients who achieved 90% recovery of TOF ratio within 5 min of study drug administration. Patients were included in the per-protocol analysis if the primary outcome measure was available and if the TOF count was truly 0 at the time of study drug administration. In the intention-to-treat analysis, all patients were included even if the TOF count was more than 0. The choice of the non-inferiority margin was done according to the division of Anti-Infective Drug Products of the FDA published recommendations in 1992 for the selection of deltas for non-inferiority trials; one approach to setting the lower bound of the 95% CI (the delta) would be to base this limit on the success rate achieved in the trial. The recommendations were for use of 10%, 15%, and 20% deltas in trials with success rates of ≥90%, 80% - 89% and ≤79%, respectively [11]. The primary outcome was expressed as numbers and percentages. Confidence intervals (CI) for difference of percentages were calculated using Wilson’s procedure without continuity correction (http://vassarstats.net/prop2_ind.html) [12]. The criterion for non-inferiority was considered to have been met if the lower limit of a 2-sided 95% CI for the difference between groups was less than 10%. For secondary outcomes, normally distributed data were reported as means ± SD and confidence intervals of the differences and analyzed using Student *t* test with equal variances. *P* < 0.05 was considered significant. The data were tested for normality using the Kolmogorov–Smirnov normality test with Lilliefors correction and were considered normally distributed if *P* > 0.05. All analyses were performed using SPSS (version 22, Chicago, IL).

Power analysis was based on non-inferiority. The results from a previous study showed that 98% of patients recover to TOF of 0.9 within 5 min after reversal of moderate rocuronium-induced NMB with sugammadex [2]. Assuming that 88% of patients would recover to TOF of 0.9 in the NS group, and a non-inferiority margin set at 10%, a sample size of 25 in each group was calculated, with α = 0.025 (one sided) and β = 0.2. To account for 10% dropout rate, we enrolled 56 patients.

## Results

**Fig. 1:**
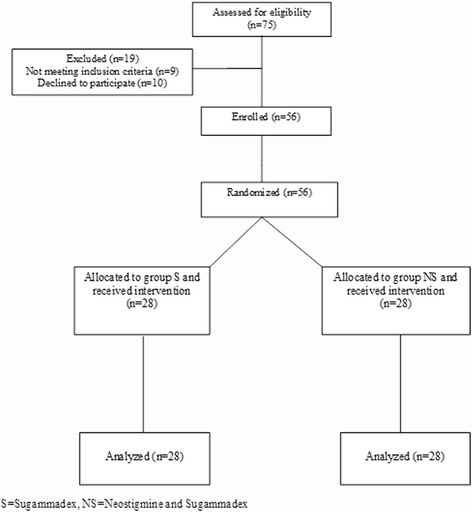
Flow chart

Fifty six patients fulfilled the inclusion criteria and signed the informed consent during the study period (February 2015-November 2015); 28 patients were enrolled in each group. No patients were excluded. All patients had a TOF of 0 before reversal administration except for three patients in group NS and 2 patients in group S who had one twitch. This twitch reappeared at the time of reversal administration. Therefore, the 5 patients were given the reversal as for deep blockade and were considered as protocol violations. They were included in the intention-to-treat analysis but not in the per-protocol analysis (Fig. 1).

**Fig. 2:**
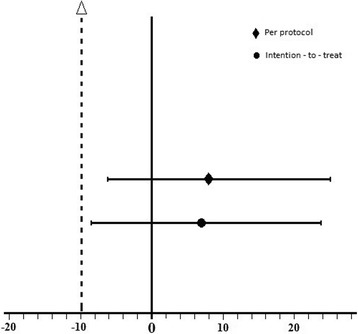
Non-inferiority limit and 95% CI of the point estimate for the percent of patients who recovered TOF ratio of 0.9 within 5 min

Operations were a mix of orthopedic, laparoscopic, gynecological, urological, head and neck, and general surgical procedure. The characteristics of patients, anesthesia and surgery were comparable between the 2 groups (Table 1). An intention-to-treat analysis showed that the number of patients who reached TOF of 0.9 within 5 min was 27 out of 28 (96%) in group S versus 25 out of 28 (89%) in group NS (difference: 7%, 95% CI of the difference: −9% to 24%). A per-protocol analysis by excluding patients with TOF of 1 showed that the number of patients who reached 90% TOF ratio within 5 min was 26 out of 26 (100%) in group S versus 23 out of 25 (92%) in group NS (difference: 8%, 95% CI of the difference: −6% to 25%) (Fig. 2). The time to achieve 90% TOF ratio was 180.9 ± 96.8 s in group S and 228.2 ± 83.9 s in group NS (*P* = 0.06, difference: 47.3 s, 95% CI of the difference: −95.9 to 1.2 s). The time from reversal to extubation was 504 ± 186 s in group S and 544 ± 176 s in group NS (*P* = 0.5, difference: 29.6 s, 95% CI of the difference: −126 to 66.8 s) (Table 2). All patients recovered 90% TOF ratio within 10 min and no rescue sugammadex was needed.

**Table 1:** Characteristics of patients and surgery

	Group S (n = 28)	Group NS (n = 28)
Age (years)	42.4 ± 14.3	40.6 ± 13.5
Gender (M/F)	15/13	16/12
Weight (Kg)	76.7 ± 18.8	72.9 ± 11.2
Height (cm)	168.3 ± 13.6	166.3 ± 12.1
BMI (Kg/m^2^)	26.9 ± 5.7	26.9 ± 6.2
ASA Status 1 2 3	14 11 3	13 13 2
Surgery time (min)	109.1 ± 84.2	100.0 ± 48.9
Anesthesia time (min)	142.4 ± 91.8	133.0 ± 52.7
Total rocurunium (mg)	104.6 ± 61.5	93.8 ± 21.1
Total rocuronium/h (mg/h)	76.6 ± 45.5	65.8 ± 35.9
Total rocuronium/Kg (mg/Kg)	1.4 ± 0.8	1.3 ± 0.4
Smax (mA)	38.0 ± 14.6	39.9 ± 10.9
Temperature at end of surgery (°C)	36.1 ± 0.5	35.8 ± 0.6

Data are means ± SD or numbers

*S* Sugammadex, *NS* Neostigmine and Sugammadex, *M* Male, *F* Female, *Smax* Supramaximal current

**Table 2:** Characteristics of neuromuscular block recovery

	Group S (n = 28)	Group NS (n = 28)	Difference	95% confidence interval of the difference
Number of patients with TOF 0.9 within 5 min (intention-to-treat analysis)	27 (96)	25 (89)	7%	−9% to 24%
Number of patients with TOF 0.9 within 5 min (per-protocol analysis)^a^	26 (100)	23 (92)	8%	−6% to 25%
Time to TOF 0.9 (sec)	180.9 ± 96.8	228.2 ± 83.9	−47.32	−95.9 to 1.2
TOF ratio at 5 min	0.96 ± 0.11	0.94 ± 0.07	0.02	−0.03 to 0.07
Time to extubation (sec)	502.5 ± 192.0	532.1 ± 167.1	−29.6	−126.0 to 66.8
Vials of sugammadex used per patient	2 ± 0.5	1.2 ± 0.4	0.82	0.59 to 1.1

The primary outcome was tested for non-inferiority and expressed as numbers (%) and confidence intervals for the difference of percentages. The non-inferiority margin delta was set at 10%. Non-inferiority was met since the lower limit of a 2-sided 95% confidence interval is less than 10%. Secondary outcomes were expressed as means ± SD and analyzed using Student *t* test

*S* Sugammadex, *NS* Neostigmine and Sugammadex, *TOF* Train of Four

^a^Per-protocol analysis: n = 26 in group S and n = 25 in group NS

SBP values were comparable between the 2 groups at all time points. HR values were comparable before and after 5 min of reversal administration. However, HR after 1 min of reversal was higher in group NS than group S (Table 3). The number of sugammadex vials per patient was 2 ± 0.5 in group S and 1.2. ± 0.4 in group NS, *P* < 0.001, difference: 0.82 vials, 95% CI of the difference: 0.59 to 1.1 vials). All patients were discharged from PACU after one hour. No signs of recurarization or adverse events were recorded.

**Table 3:** Hemodynamic changes after administration of reversal agent

	Group S (n = 28)	Group NS (n = 28)	*P*	Difference	95% confidence interval of the difference
HR before reversal	65.4 ± 13.1	68.4 ± 12.2	0.37	−3.03	−9.8 to 3.8
HR after 1 min	64.7 ± 15.3	74.5 ± 17	0.03	9.82	−18.5 to −1.1
HR after 5 min	70.7 ± 18.4	70.5 ± 17.6	0.95	0.28	−9.4 to 10
SBP before reversal	118.6 ± 20.5	117.8 ± 14.9	0.88	0.75	−8.8 to 10.3
SBP after 1 min	121 ± 17.1	127.1 ± 22.2	0.26	−6.03	−16.7 to 4.6
SBP after 5 min	123.9 ± 20	125.4 ± 24.7	0.8	−1.5	−13.4 to 10.4

Data were expressed as means ± SD and analyzed using Student *t* test

*S* Sugammadex, *NS* Neostigmine and Sugammadex, *HR* Heart Rate, *SBP* Systolic Blood Pressure

## Discussion

We showed that sugammadex 2 mg/kg with neostigmine 50 μg/kg is non-inferior to sugammadex 4 mg/kg in reversing deep NMB, as per-protocol and intention-to-treat analyses, with a similar proportion of patients recovering 90% TOF ratio within 5 min in both groups. The times to 90% recovery of TOF ratio and to extubation were comparable between the 2 groups. No cases of recurarization were detected in the PACU. A moderate and transient increase in HR was detected in the combination group.

Sugammadex reverses rocuronium blockade in a dose-dependent manner [13] and achieves full recovery more rapidly than neostigmine [3]. Applying half the recommended dose to reverse deep NMB (2 mg/kg in group NS) brought the patient into a shallower level of blockade and the reversal action was efficiently completed with neostigmine. The speed of action of sugammadex offers a major advantage over neostigmine reversal which might take up to 15 min to achieve its peak effect [14]. This advantage was not lost in the combination group, since approximately 90% of patients achieved full recovery within only 5 min, and 100% achieved full recovery in less than 10 min with no signs of rNMB. Despite the fact that the full effect of neostigmine may take more than ten minutes, its effect could start earlier. The onset of action of action of neostigmine starts after 2 min [14]. Moreover, in a previous study Schaller et al. [9] reported that 0.22 mg/kg suggamadex and 34 μg/kg neostigmine accelerates recovery from a TOF ratio of 0.5 to a TOF ratio of at least 0.9 in an average of 2 min but within 5 min for 95% of all treated patients.

Eikermann et al. [15] showed that the administration of neostigmine in the absence of residual NMB may impair genioglossus and diaphragm muscle function. Furthermore, Cammu et al. [16] showed that the administration of neostigmine following 2 mg/kg of sugammadex to reverse moderate neuromuscular blockade resulted in a weaker diaphragmatic electromyographic activity than sugammadex alone. However, in our study no rNMB blockade was detected neither clinically nor per the TOF. This might be explained by the fact that we administered only half the recommended dose of sugammadex which is not expected to produce complete recovery from deep NMB.

Sugammadex was used as a rescue drug in a patient who didn’t return to full recovery after neostigmine [17]. Hence, the idea of sugammadex use for partial paralysis following insufficient recovery by neostigmine was raised. Subsequently, sugammadex was used as a rescue drug for incomplete reversal of rocuronium-induced NMB after neostigmine in a renal failure patient [18]. In a recent randomized trial, Cheong and colleagues investigated the combination of low dose sugammadex and neostigmine for reversal of rocuronium-induced moderate NMB [19]. The authors compared the time to 90% TOF ratio recovery in 4 reversal groups: sugammadex 2 mg/kg (S2), sugammadex 1 mg/kg (S1), sugammadex 1 mg/kg + neostigmine 50 μg/kg (SN) and neostigmine 50 μg/kg alone (N). At the time of reversal, TOF count was 1 or 2. The total amount of rocuronium received per patient was not reported. Group SN showed significantly shorter recovery time than group S1 and N. Similar to our study, no statistically significant differences between the S2 and SN groups were observed. Furthermore, the recovery time from moderate NMB of 183 s in group S2 and 204 s in group SN were similar to the recovery times from deep NMB in our study (181 s. in group S and 228 s. in group NS). Our study is the first to assess recovery from deep NMB with half dose sugammadex and neostigmine. Only sugammadex ampoules of 200 mg are available. Therefore, the combination is not a useful cost containment strategy for the reversal of moderate NMB as in Cheong and colleagues, since using 1 mg/kg or 2 mg/kg would both result in opening one ampoule in an average 70 kg patient. Conversely, in our study, we demonstrated a substantial cost saving with the multimodal reversal from deep NMB. Using 2 mg/kg sugammadex instead of 4 mg/kg resulted in an approximate reduction of 50% in the number of vials used, i.e. using 1 vial instead of 2 vials for patients who weigh 51–100 kg. Other than the depth of the NMB, our methodology was different from the above mentioned study. We used a non-inferiority design because it is more specific to our hypothesis. Also, we set the primary outcome as the percentage of patients who achieved full recovery within 5 min rather than the absolute recovery time value. We considered this primary outcome more meaningful because of the importance of shortening the residual paralysis period between the administration of the reversal and the return to 90% TOF ratio. Most of our patients in the combination group achieved full recovery within 5 min and preserved the characteristics of sugammadex recovery [2] despite the administration of approximately 0.8 mg/kg/h of rocuronium and the maintenance of a deep level of NMB till the end of surgery.

The high cost of sugammadex has so far prevented its routine use. Ledowski and colleagues found that the cost of muscle relaxants and reversal increased from 42$ to 127$ per case after the introduction of unrestricted use of sugammadex [20]. Fuchs-Buder and colleagues assessed the feasibility of the routine use of sugammadex for reversal of rocuronium-induced NMB [21]. They concluded that despite the potential improvement in recovery times, its routine use could not be justified. In many institutions, restrictive strategies are adopted to decrease the use of expensive medications. A significant anesthesia cost-reduction and decreased utilization of cost-prohibitive agents was demonstrated after applying accessibility interventions [22]. More physicians have the tendency to use sugammadex when it is available and easily reachable. A retrospective study showed a significant increase of sugammadex use when its availability became unrestricted [23]. Should we find ways to cut down on the high cost of this drug, its use is expected to grow up significantly. In the present study, we introduced an approach that significantly decreased the cost of reversal without losing the benefits.

Sugammadex does not interact with the cholinergic system and consequently is not accompanied by the cardiovascular adverse effects that are seen with acetylcholinesterase inhibitors. In our study, the cardiovascular changes that followed neostigmine/glycopyrrolate administration were of limited amplitude and of small clinical significance in a relatively healthy population.

One limitation of our study is the inability to extrapolate our results to situations where sugammadex administration might be of a great help (bariatric surgeries). Of note, case of recurarization after administration of sugammadex for moderate NMB reversal in an obese patient was reported postoperatively [24]. Since sugammadex is not well studied in the obese population, we excluded patients with BMI more than 35 kg/m^2^. Therefore, the generalizability of our results couldn’t be extended to obese patients. Future studies in bariatric surgery might be needed. Another limitation is the relatively long time to extubation of approximately 500 s in both groups. This delay between 90% TOF ratio and extubation occurred because we kept an adequate depth of anesthesia by using dexmedetomidine during the recovery phase in order to secure motionless hands during TOF measurements. A third limitation is that the cost savings on sugammadex do not apply automatically and depend on the local situation in the single countries.

## Conclusion

In conclusion, we demonstrated that the combination of neostigmine 50 μg/kg and sugammadex 2 mg/kg is at worse 9% and 6% less effective than sugammadex 4 mg/kg by intention-to-treat and per-protocol analysis respectively, in terms of the proportion of patients who achieved full recovery from rocuronium-induced deep NMB within 5 min. Thus, the combination of neostigmine and half dose sugammadex is non-inferior to full dose sugammadex. Anesthesiologists might face the need to reverse deep NMB because of unanticipated short surgeries, inter-individual variability in the response to non-depolarizing muscle relaxants or as rescue for incomplete neostigmine reversal. Although cost price should not outweigh good clinical practice, our study has identified a safe cost saving strategy for the use of sugammadex for these clinical scenarios.

## Abbreviations

NMB: Neuromuscular blockade
TOF: Train of Four
SBP: Systolic blood pressure
HR: Heart rate
PACU: Post anesthesia care unit
rNMB: Residual neuromuscular blockade
